# The Antecedents and Consequences of Extended Nursing Staff Work Hours in Nursing Homes

**DOI:** 10.1093/geroni/igaf122.1494

**Published:** 2025-12-31

**Authors:** Rohit Pradhan, Robert Weech-Maldonado

## Abstract

A key determinant of nursing home (NH) performance, particularly quality, is the adequacy and the expertise of nursing staff [registered (RNs), licensed practical nurses (LPNs), and certified nursing assistants (CNAs)]. Nursing staff serve as primary caregivers in NHs, directly influencing resident care and outcomes. Yet, burnout remains a significant concern, and NHs experience extraordinary levels of turnover. Extended work hours among nursing staff may be an important factor contributing to this challenge. While existing research shows that prolonged work hours can negatively affect healthcare workers’ well-being—leading to poorer quality outcomes and higher turnover rates—little is known about how extended nursing staff work hours specifically affect NH outcomes. NHs differ substantially from acute care settings for various reasons, such residents’ need for sustained and continuous care. Therefore, it is important to understand the implications of extended work hours on resident and staff outcomes in NHs. The overarching goal of this symposium is to explore the antecedents and consequences of extended work hours nursing staff in NHs. As part of the symposium, we have included studies examining how extended work hours relate to quality, turnover, the organizational and environmental factors associated with extended work hours, and overall NH costs. The findings and discussions may offer valuable insights for managerial decision-making and policy and regulatory formulation. They can help address the systemic challenges of nursing staffing in NHs and improve NH performance, particularly regarding quality of care and turnover.

